# Editorial: Methods in pediatric infectious diseases 2022

**DOI:** 10.3389/fped.2023.1304163

**Published:** 2023-11-02

**Authors:** Yue Wang, Camille Bréhin, Zhongjie Shi

**Affiliations:** ^1^ Department of Pharmacology & Toxicology, Wright State University, Dayton, OH, United States; ^2^ Service d'accueil des Urgences Pédiatriques, Hôpital des Enfants, Centre Hospitalier Universitaire de Toulouse, Toulouse, France; ^3^ Department of Pediatrics, Wayne State University, Detroit, MI, United States

**Keywords:** pediatrics—children, infectious diseases, methods, challenges, tool

**Editorial on the Research Topic** Methods in pediatric infectious diseases 2022

Pediatric infectious diseases encompass a wide range of bacterial, viral, and fungal infections, and can be latent as early as during pregnancy (1–4). As a result, the methodologies employed to investigate the underlying mechanisms and clinical data exhibit significant diversity. Within this special issue, we are pleased to present four distinct studies, each offering compelling and thought-provoking findings.

Rotavirus infection is a known risk factor for type 1 diabetes (T1D) and celiac disease (CD). By conducting a systematic review and meta-analysis, Zhang et al. comprehensively evaluated the association between rotavirus vaccination and the risks of T1D or CD. They found that rotavirus vaccination does not alter the subsequent risk of T1D or CD. However, children vaccinated below 5 years old exhibited a lower risk for T1D than older children, suggesting that the protective effect of rotavirus vaccination on T1D may be influenced by the timing of vaccination.

Epstein-Barr virus (EBV) is a type of human lymphotropic herpes virus that primarily affects children and adolescents. Most cases of EBV infection in this age group are asymptomatic or present with atypical symptoms. In the study conducted by Yang and Zhu, the status of EBV infection and influencing factors among children were analyzed using a combined detection method involving specific antibodies and DNA. The number of siblings and exposure to family members who smoke were identified to be independent risk factors for EBV infection. Conversely, parents with a higher level of education, longer duration of breastfeeding, and the use of dedicated tableware were identified to be independent protective factors. This article improves our understanding of the spread of EBV infection.

Pneumonia has remained the leading cause of mortality in young children for a considerable period. Xu et al. employed a nomogram prediction model to analyze the clinical features and risk factors for poor outcomes of severe community-acquired pneumonia (SCAP) in children. This approach allows for a more personalized and precise assessment of individual patients. By incorporating this predictive tool into clinical practice, healthcare professionals can make informed decisions regarding the management and treatment of children with SCAP, ultimately improving patient outcomes.

In resource-limited settings, the availability of pulse oximeters can be limited. To address this challenge, Schuh et al. developed clinical scores to identify hypoxemia in children with pneumonia based on two outpatient datasets of 3–35-month-old children with pneumonia in Bangladesh and Malawi. The authors found that those scores were more potent to identify outpatient hypoxemic pneumonia cases than the WHO guidelines. The use of the clinical score can be used by healthcare professionals with minimal training.

The challenging tasks for the methods in pediatric infectious diseases include the in-time update of the changing spectrum of diseases, the advances in understanding the mechanism of diseases, translational models, mathematical and statistical tools, online diagnosis, data mining, and the fast progress in vaccines, as exemplified by the Covid-19 pandemic.
